# Assessing coronary artery stenosis exacerbated impact on left ventricular function and deformation in metabolic syndrome patients by 3.0 T cardiac magnetic resonance imaging

**DOI:** 10.1186/s12933-024-02492-9

**Published:** 2024-11-18

**Authors:** Yi-Ning Jiang, Yue Gao, Chen-Yan Min, Ying‑Kun Guo, Rong Xu, Li-Ting Shen, Wen-lei Qian, Yuan Li, Zhi-Gang Yang

**Affiliations:** 1 Department of Radiology, West China Hospital, Sichuan University, 37# Guo Xue Xiang, Chengdu, 610041 Sichuan China; 2 Department of Radiology, Key Laboratory of Obstetric and Gynecologic and Pediatric Diseases and Birth Defects of Ministry of Education, West China Second University Hospital, Sichuan University, Chengdu, 610041 China

**Keywords:** Coronary artery stenosis, Metabolic syndrome, Global peak strain, Cardiac magnetic resonance imaging

## Abstract

**Background:**

Metabolic syndrome (MetS) and coronary artery stenosis (CAS) independently increase the risk of cardiovascular events, while the impact of CAS on left ventricular (LV) function and deformation in MetS patients remains unclear. This study investigates how varying degrees of CAS exacerbate LV function and myocardial deformation in MetS patients.

**Methods:**

One hundred thirty-one MetS patients who underwent CMR examinations were divided into two groups: the MetS(CAS−) group (n = 47) and the MetS(CAS+) group (n = 84). The MetS(CAS+) group was divided into MetS with non-obstructive CAS(NOCAS+) (n = 30) and MetS with obstructive CAS(OCAS+) group (n = 54). Additionally, 48 age- and sex-matched subjects were included as a control group. LV functional and deformation parameters were measured and compared among subgroups. The determinants of decreased LV global peak strains in all MetS patients were identified using linear regression. The receiver operating characteristic (ROC) curve and logistic regression model (LRM) evaluated the diagnostic accuracy of the degree of CAS for identifying impaired LV strain.

**Results:**

Compared to MetS(CAS−), MetS(NOCAS+) showed a significantly increased LV mass index (p < 0.05). Global longitudinal peak strain was decreased gradually from MetS(CAS−) through MetS(NOCAS+) to MetS(OCAS+) (− 13.02 ± 2.32% vs. − 10.34 ± 4.05% vs. − 7.55 ± 4.48%, p < 0.05). MetS(OCAS+) groups showed significantly decreased LV global peak strain (GPS), PSSR and PDSR in radial and circumferential directions compared with MetS(NOCAS+) (all p < 0.05). The degree of CAS was independently associated with impaired global radial peak strain (GRPS) (β =  − 0.289, p < 0.001) and global longitudinal peak strain (GLPS) (β = 0.254, p = 0.004) in MetS patients. The ROC analysis showed that the degree of CAS can predict impaired GRPS (AUC = 0.730) and impaired GLPS (AUC = 0.685).

**Conclusion:**

Besides traditional biochemical indicators, incorporating CAS assessment and CMR assessment of the LV into routine evaluations ensures a more holistic approach to managing MetS patients. Timely intervention of CAS is crucial for improving cardiovascular outcomes in this high-risk population.

**Supplementary Information:**

The online version contains supplementary material available at 10.1186/s12933-024-02492-9.

## Introduction

Metabolic syndrome (MetS) is a complex disorder characterized by a cluster of interconnected risk factors, including abdominal obesity, insulin resistance, hypertension, and dyslipidemia [1]. The escalating prevalence of MetS has underscored its status as a pressing public health issue [2]. Among the cardiovascular complications associated with MetS, coronary artery disease (CAD) stands out as a leading cause of morbidity and mortality worldwide. The incidence and mortality of cardiovascular complications were 1.83 (1,000 person-year) and 1.75 (1,000 person-year) in MetS patients, with CAD mortality accounting for approximately 34.04% of cardiovascular complications [3, 4]. MetS is believed to contribute to the development and progression of atherosclerosis through mechanisms involving chronic inflammation, endothelial dysfunction, and dysregulated lipid metabolism, leading to increased atherosclerotic plaque in coronary arteries and microcirculation [5, 6]. As a hallmark feature of CAD, coronary artery stenosis (CAS) compromises myocardial perfusion and can lead to myocardial ischemia, impaired left ventricular (LV) function, and major adverse cardiovascular events (MACE) [7, 8]. Additionally, studies have shown that in the early stage of MetS, patients with apparently normal or nearly normal coronary arteries, as revealed by coronary angiography, may still experience coronary microvascular dysfunction and remodeling [9, 10]. In this context, the presence of CAS may exacerbate the myocardial damage of MetS patients, leading to a significantly increased risk of MACE in MetS patients, even in the non-obstructive CAS (NOCAS) stage [11].

However, due to the unique and complex pathophysiological profile of MetS, the additive effect of varying degrees of CAS on LV function and deformation in MetS patients is still not fully clear. LV deformation, especially longitudinal peak strain, is linked to a higher risk of MACE, offering critical insights for predicting cardiovascular outcomes [12]. Therefore, understanding myocardial impairment related to CAS in MetS patients is crucial for early prevention and deceleration of the progression of their condition. Previous studies have assessed LV function and strain impairment in patients with MetS using echocardiography-based myocardial measurements [13]. Cardiac magnetic resonance (CMR) provides comprehensive information for detailed imaging of cardiac structures, function, and evaluation of myocardial strain [14, 15]. Therefore, this study aimed to investigate the exacerbated impact on LV function and LV myocardial strain related to varying degrees of CAS, assessed by coronary angiography in MetS using CMR and to explore further the independent determinants of LV global peak strain (GPS) decline in patients with MetS.

## Materials and methods

### Study population

The study protocol was approved by the West-China Hospital of Sichuan University Biomedical Research Ethics Committee, and due to the retrospective nature of the analyses, no written informed consent was required.

We retrospectively collected hospitalized patients who underwent CMR scans from January 2013 to October 2023. The diagnosis criteria for MetS followed the 2009 Joint Interim Statement of the International Diabetes Federation Task Force on Epidemiology and Prevention [16]. The presence of any 3 of the five risk factors constitutes the diagnosis of MetS: (1) elevated waist circumference(WC, ≥ 85 cm in males and ≥ 80 cm in females) or body mass index (BMI) > 25 kg/m^2^ [17]; (2) elevated triglycerides (TG) {≥ 150 mg/dL (1.7 mmol/L)} or drug treatment for elevated TG; (3) reduced high-density lipoprotein cholesterol (HDL-C) {< 40 mg/dL (1.0 mmol/L) in males; < 50 mg/dL (1.3 mmol/L) in females} or drug treatment for reduced HDL-C; (4) elevated blood pressure (systolic ≥ 130 and/or diastolic ≥ 85 mm Hg) or antihypertensive drug treatment in a patient with a history of hypertension; (5) fasting glucose ≥ 100 mg/dL or drug treatment of elevated glucose. Exclusion criteria: (1) received interventions affecting coronary artery evaluation (including revascularization, stent implantation, prosthetic valve replacement before CMR); (2) congenital heart disease, primary cardiomyopathy, myocardial infarction, moderate to severe valvular abnormalities, and so on; (3) incomplete critical clinical information; and (4) severe artifacts, or poor image quality for evaluation.

Patient demographics, clinical history, cardiovascular risk factors, and lab results were documented using hospital and laboratory information management systems. Hypertension was defined as systolic blood pressure (SBP) ≥ 140 mmHg and/or diastolic blood pressure (DBP) ≥ 90 mmHg confirmed by three separate measurements on different days or by a prior diagnosis of essential hypertension or current use of antihypertensive medication [18]. Type 2 diabetes mellitus (T2DM) adhered to the American Diabetes Association guidelines or was presently managed with oral glucose-lowering medications or insulin therapy [19]. Regardless of whether they had quit smoking or not, they were all recorded as having a history of smoking. Obesity was defined as BMI ≥ 25 kg/m^2^ [17]. The degree of CAS assessed by coronary angiography was divided into no CAS, NOCAS, and obstructive CAS (OCAS). OCAS is defined as ≥ 50% stenosis of any of the major epicardial coronary arteries, including the left anterior descending branch (LAD), left circumflex branch (LCX), right coronary artery (RCA), or left main (LM) coronary artery, otherwise, it is defined as NOCAS [20]. Record the most severe stenosis any of the major epicardial coronary arteries for each patient. The overall severity of CAS was assessed using the Gensini score [21].

Age- and sex-matched subjects who underwent CMR were included as the control group with no history of impaired fasting glucose, hypertension, obesity, electrocardiogram abnormalities, symptoms of cardiovascular disease, or cardiovascular abnormalities detected using CMR {reduced ejection fraction (EF) in both ventricles, abnormal ventricular motion, valvular stenosis, or regurgitation, etc.}.

### CMR protocols

All participants underwent CMR using a whole-body 3.0 T scanner (MAGNETOM Skyra or Trio Tim system, Siemens Medical Solutions, Erlangen, Germany). We acquired retrospective electrocardiogram-gated cine images utilizing a balanced steady-state free-precession sequence. This imaging protocol involved obtaining a set of 8–12 continuous short-axis slices covering both ventricles from the level of the mitral valve annulus to the apex of the LV, along with four- and two-chamber long-axis views of the LV. The scanning parameters were as follows: a temporal resolution of 39.34/42 ms; a repetition time of 2.81/3.4 ms; an echo time of 1.22/1.3 ms; a flip angle of 38°/50°; a field of view of 250 × 300 mm^2^ or 340 × 285 mm^2^; a matrix size of 256 × 166/208 × 139, and a slice thickness of 8 mm. Additionally, late gadolinium enhancement (LGE) images were acquired in the same slice positions as the cine imaging, typically 10–15 min after the administration of contrast. These images were obtained using a phase-sensitive inversion recovery sequence, with parameters including a temporal time of 300 ms, TE of 1.44 ms, a flip angle of 40°, a slice thickness of 8 mm, a field of view of 275 × 400 mm^2^, and a matrix size of 256 × 184.

### CMR measurements

For measurement, all CMR images were uploaded to offline commercial software (Cvi42, Circle Cardiovascular Imaging, Inc., Calgary, Canada). For the analysis of LV functional parameters, an artificial intelligence-based tool automatically delineated the contours of the endocardium and epicardium of the LV in the end-diastolic and end-systolic phases from the stack of short-axis movie images. Then, a researcher manually adjusted the contours and confirmed the accurate phase, and the software automatically calculated the LV end-diastolic volume (LV-EDV) and LV end-systolic volume (LV-ESV), LV stroke volume (LV-SV), LV mass (LVM) and LVEF. The LVEDV, LVESV, and LVM were all standardized by body surface area [22]. The LV short-axis and LV long-axis slices (2-chamber and 4-chamber) were input to the feature-tracking module to analyze the LV myocardial strain parameters. The LV global radial (LV-GRPS), circumferential (LV-GCPS) longitudinal peak strain (LV-GLPS), and peak systolic strain rate (PSSR) and peak diastolic strain rate (PDSR) were also calculated automatically. The LV global function index (LVGFI) was calculated using the following formula [23]: LVGFI = {LVSV/[(LVEDV + LVESV)/2 + (LVM/1.05)]} × 100.

LGE was considered positive (LGE+) if hyperintense regions were observed within the myocardium at the short- and long-axis views. Two radiologists categorized LGE distributions into five categories: (1) None; (2) subendocardial (3) midmyocardial (4) subepicardial (5) transmural [24]. Both observers (Y.N.J., a cardiothoracic radiologist with five years of experience; Y.G., a cardiothoracic radiologist with nine years of experience) independently assessed LGE images, resolving discrepancies through discussion for consensus.

### Reproducibility of LV function and strain parameters

Intra- and inter-observer reproducibility was evaluated by randomly selecting 50 CMR images (35 MetS patients and 15 control subjects), and all the imaging analyses were completed by two experienced radiologists (Y.N.J. and Y.G.). The radiologist (Y.N.J) completed the same 50 randomly selected CMR images and all others at least one month later. Evaluate inter-observer reproducibility by comparing measurements by two observers. Evaluate intra-observer reproducibility by comparing measurements by observer one. The entire image analysis process operates independently and blindly.

### Statistical analysis

The Kolmogorov–Smirnov test assessed the normal distribution of continuous variables. Data were expressed as the mean ± standard deviation or medians with interquartile ranges (IQRs) for continuous variables and frequencies for categorical variables. One-way ANOVA determined differences of continuous variables among multiple groups, followed by the Kruskal–Wallis rank test or Bonferroni’s post hoc test, as appropriate. For differences between the two groups, the Student’s *t*-tests were used for normally distributed continuous variables, and Mann–Whitney U tests were used for non-normally distributed variables. Pearson *Χ*^2^ test and Fisher’s exact test for categorical variables. Univariable linear regression analysis explored the associations between LV function and strains and other factors in MetS patients. Candidate variables with no collinearity and p < 0.05 were included in the multivariable linear stepwise regression model to identify the effects of the degree of CAS and clinical factors on LV strains in MetS patients. Furthermore, MetS patients were stratified according to median value of GRPS and GLPS. The value < 26.75% was defined as impaired GRPS. The value >  − 11.08% was defined as impaired GLPS. Variables with p < 0.05 in multiple linear regression were used to construct a logistic regression model (LRM), and receiver operating characteristic (ROC) curve analysis were performed to quantify the diagnostic efficiency of the degree of CAS and other factors for impaired LV strain parameters. Intraclass correlation coefficients (ICCs) were used to evaluate intra- and inter-observer reproducibility. A two-tailed p-value < 0.05 indicated statistical significance. All statistical analyses were performed using SPSS (version 26.0, IBM, Armonk, NY, USA). The production of all violin plots was completed using GraphPad Prism (version 9.5.0).

## Results

### Demographic and clinical characteristics

A total of 131 MetS patients were included, divided into the MetS(CAS−) group (n = 47, mean age 52 ± 14 years, 26 males) and the MetS(CAS +) group (n = 84, mean age 60 ± 9 years, 65 males). In addition, 48 subjects (mean age 58 ± 8 years, 34 males) were included in the control group. The main clinical baseline characteristics of the study cohort are summarized in Table 1. From the control group through the MetS(CAS−) to the MetS(CAS +) groups, BMI significantly increased, with pairwise differences (23.41 ± 2.97 kg/m^2^ vs. 26.26 ± 2.93 kg/m^2^ vs. 26.45 ± 3.13 kg/m^2^, p < 0.001). SBP and DBP increased in both the MetS(CAS−) and MetS(CAS+) groups compared to that in the control group (all p < 0.05). Patients in the MetS(CAS+) group had a significantly higher prevalence of hypertension (92.86% vs. 63.83%, p < 0.05) and T2DM (55.95% vs. 34.04%, p < 0.05) compared to MetS(CAS−) group. Compared with the MetS(CAS−) group, the MetS(CAS+) group presented elevated levels of HbA1c, TG, HDL, troponin, NT-proBNP, and reduced eGFR (all p < 0.05).

**Table 1:** Demographic and clinical characteristics of the study participants

	Control(n = 48)	MetS (n = 131)	
		CAS− (n = 47)	CAS+ (n = 84)
Baseline characteristics			
Age (years)	58 ± 8	52 ± 14^*^	60 ± 9^§^
Male, n (%)	34 (70.83%)	26 (55.32%)	65 (77.38%) ^§^
BMI (kg/m^2^)	23.41 ± 2.97	26.26 ± 2.93^*^	26.45 ± 3.13^*§^
Smoking, n (%)	–	14 (29.79%)	27 (32.14%)
SBP (mmHg)	128 ± 17	133 ± 23^*^	138 ± 21^*^
DBP (mmHg)	77 ± 9	84 ± 18	84 ± 14^*^
HR, min^−1^	73 ± 11	75 ± 16	77 ± 15
Cardiovascular risk (n, %)			
Hypertension	–	30 (63.83%)	78 (92.86%)^§^
T2DM	–	16 (34.04%)	47 (55.95%)^§^
Dyslipidemia	–	44 (93.62%)	72 (85.71%)
Obesity	–	32 (68.09%)	56 (66.67%)
Laboratory parameters			
HbA1c (%)	–	6.40 ± 0.87	7.27 ± 1.35^§^
TG, mmol/L	–	2.74 ± 1.76	2.59 ± 2.03^§^
TC, mmol/L	–	4.26 ± 1.19	3.96 ± 1.13^§^
HDL, mmol/L	–	0.84 ± 0.27	0.92 ± 0.25^§^
LDL, mmol/L	–	2.28 ± 0.88	2.24 ± 0.89
Troponin, ng/L	–	70.30 ± 166.08	150.38 ± 400.30^§^
NT-proBNP	–	444.27 ± 843.91	1279.96 ± 1997.07^§^
eGFR (mL/min/1.73m^2^)	–	77.20 ± 28.66	73.20 ± 22.59^§^
Concomitant medication, n (%)			
Insulin	–	5 (10.64%)	13 (15.48%)
Biguanides	–	2 (4.26%)	19 (22.62%)^§^
ACEI/ARB	–	12 (25.53%)	35 (41.67%)
Satins	–	4 (8.51%)	22 (26.19%)^§^
Aspirin	–	3 (6.38%)	24 (28.57%)^§^
Coronary artery related parameters			
Number of coronary arteries affected, n (%) One/Two/Three-vessel	–	–	15 (17.85%)/21 (25.00%)/48 (57.15%)
Location of coronary artery occlusion, n (%) RCA /LM/LAD/LCX)	–	–	31 (36.90%)/6 (7.14%)/44 (52.38%)/31 (36.90%)
Most severe stenosis (%)	–	–	43.93 ± 41.03
Gensini score	–	–	46.42 ± 50.13

All values are presented as n (%), mean ± standard deviation. BMI, body mass index; SBP, systolic blood pressure; DBP, diastolic blood pressure; HR, heart rate; T2DM, type 2 diabetes mellitus; HbA_1_c, glycated haemoglobin; TG, triglyceride; TC, total cholesterol; HDL-C, high density lipoprotein cholesterol; LDL-C, low density lipoprotein cholesterol; NT‑proBNP, amino-terminal pro-B-type natriuretic peptide; eGFR, estimated glomerular filtration rate; ACEI, angiotensin converting enzyme inhibitor; ARB, angiotensin receptor blocker

^*^: p < 0.05 vs. Control group

^§^: p < 0.05 vs. MetS (CAS−) group

### Comparison of LV function and strain parameters among control, MetS without and with CAS

LV function and deformation parameters among control, MetS(CAS−), and MetS (CAS+) groups are shown in Table 2. Compared to the control and MetS(CAS−), the MetS(CAS+) group presented increased LV-EDVi, LV-ESVi, and LVMi, and decreased LV-SVi, LVEF, and LVGFI (all p < 0.05). LV GPS and in PSSR in all three directions, PDSR-S and PDSR-C showed significant decreased in the MetS(CAS+) group compared to both control and MetS(CAS−) groups (all p < 0.05). LV GRPS, GLPS, and PDSR in all three directions showed a significant decrease in the MetS(CAS−) group compared to the control group (GRPS, 32.03 ± 9.61% vs. 37.36 ± 6.85%; GLPS, − 13.02 ± 2.32% vs. − 15.21 ± 2.41%, all p < 0.05).

**Table 2:** Comparison of LV function and strain parameters among control group, MetS (CAS−), and MetS (CAS+) groups

	Control(n = 48)	MetS (n = 131)	
		CAS− (n = 47)	CAS+ (n = 84)
Function parameters			
LV-EDVi, mL/m^2^	70.72 ± 13.04	72.49 ± 22.21	89.79 ± 38.46^*§^
LV-ESVi, mL/m^2^	23.34 ± 5.63	26.46 ± 11.86	48.99 ± 40.15^*§^
LV-SVi, mL/m^2^	47.38 ± 8.75	46.02 ± 14.80	40.81 ± 11.18^*§^
LVMi, g/m^2^	39.76 ± 9.12	48.13 ± 16.03	63.71 ± 21.47^*§^
LVEF, %	67.08 ± 4.34	63.85 ± 11.72	52.26 ± 19.69^*§^
LVGFI	52.72 ± 5.88	49.02 ± 11.10	36.21 ± 14.71^*§^
LV strain parameters			
LV GPS, %			
GRPS, %	37.36 ± 6.85	32.03 ± 9.61^*^	21.72 ± 12.33^*§^
GCPS, %	− 21.05 ± 2.03	− 18.99 ± 5.41	− 15.12 ± 6.13^*§^
GLPS, %	− 15.21 ± 2.41	− 13.02 ± 2.32^*^	− 8.55 ± 4.53^*§^
PSSR(1/s)			
PSSR-R	2.16 ± 0.51	2.15 ± 0.94	1.37 ± 1.00^*§^
PSSR-C	− 1.08 ± 0.21	− 1.09 ± 0.39	− 0.82 ± 0.43^*§^
PSSR-L	− 0.82 ± 0.20	− 0.80 ± 0.44	− 0.50 ± 0.71^*§^
PDSR(1/s)			
PDSR-R	− 2.79 ± 1.46	− 2.09 ± 1.01^*^	− 1.42 ± 1.35^*§^
PDSR-C	1.37 ± 0.19	1.16 ± 0.41^*^	0.84 ± 0.30^*§^
PDSR-L	0.94 ± 0.47	0.69 ± 0.54^*^	0.55 ± 0.34^*^

All values are presented as mean ± standard deviation. The “ − ” indicates the direction of strains. LV, left ventricular; EDV, end-diastolic volume; ESV, end-systolic volume; EF, ejection fraction; SV, stroke-volume; M, mass; i: index; LVGFI, left ventricular global function index; GPS global peak strain; GRPS, global radial peak strain; GCPS, global circumferential peak strain; GLPS, global longitudinal peak strain; PSSR, peak systolic strain rate; PDSR, peak diastolic strain rate

^*^: p < 0.05 vs. the control group

^§^: p < 0.05 vs. MetS (CAS−) group

### Comparison of LV function and strain parameters among MetS with varying degrees of CAS

**Fig. 1:**
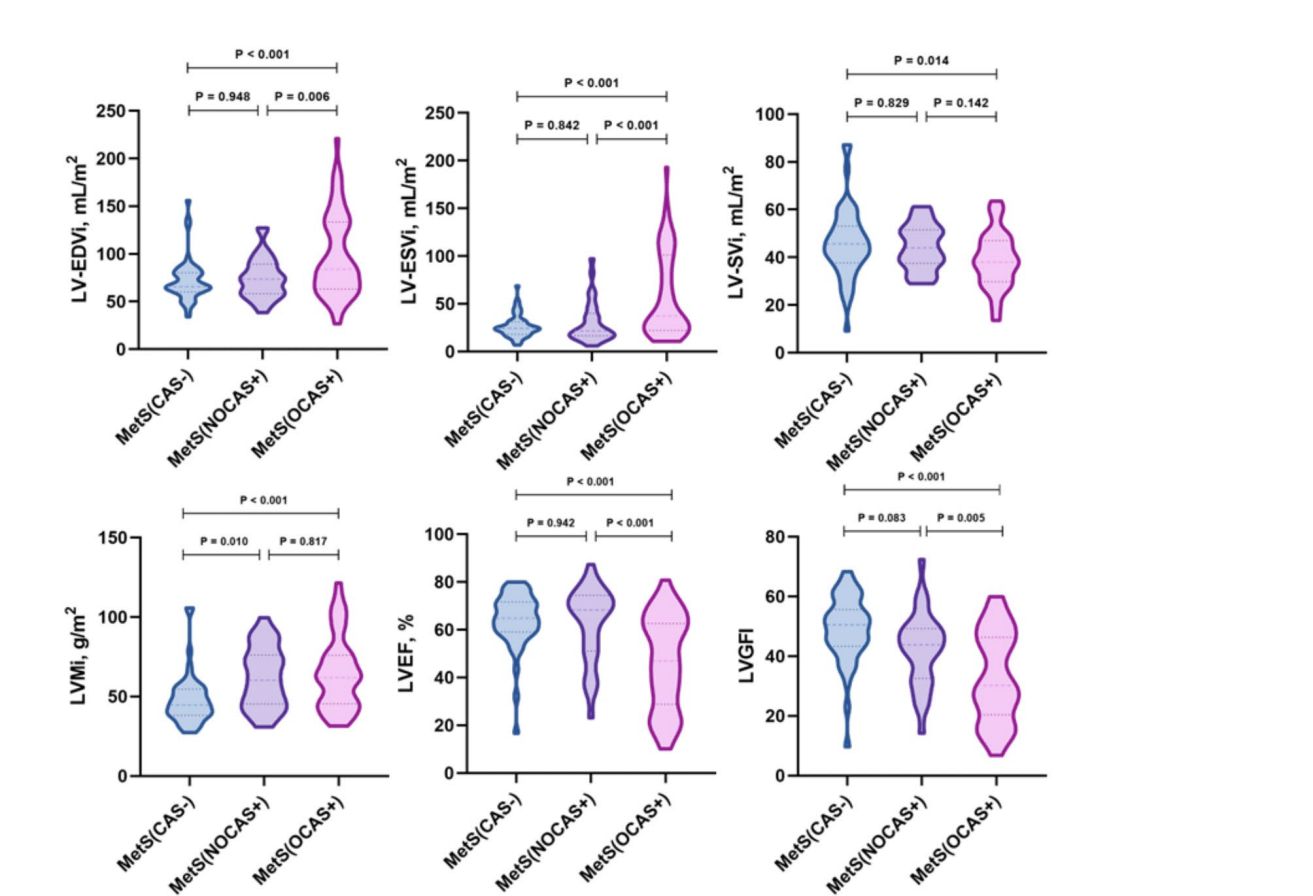
Violin plots comparing LV function parameters between MetS(CAS−), MetS(NOCAS+), MetS(OCAS+) groups. LV, left ventricular; EF, ejection fraction; EDV, end-diastolic volume; ESV, end-systolic volume; SV, stroke-volume; M, mass; i: index; LVGFI, left ventricular global function index

**Fig. 2:**
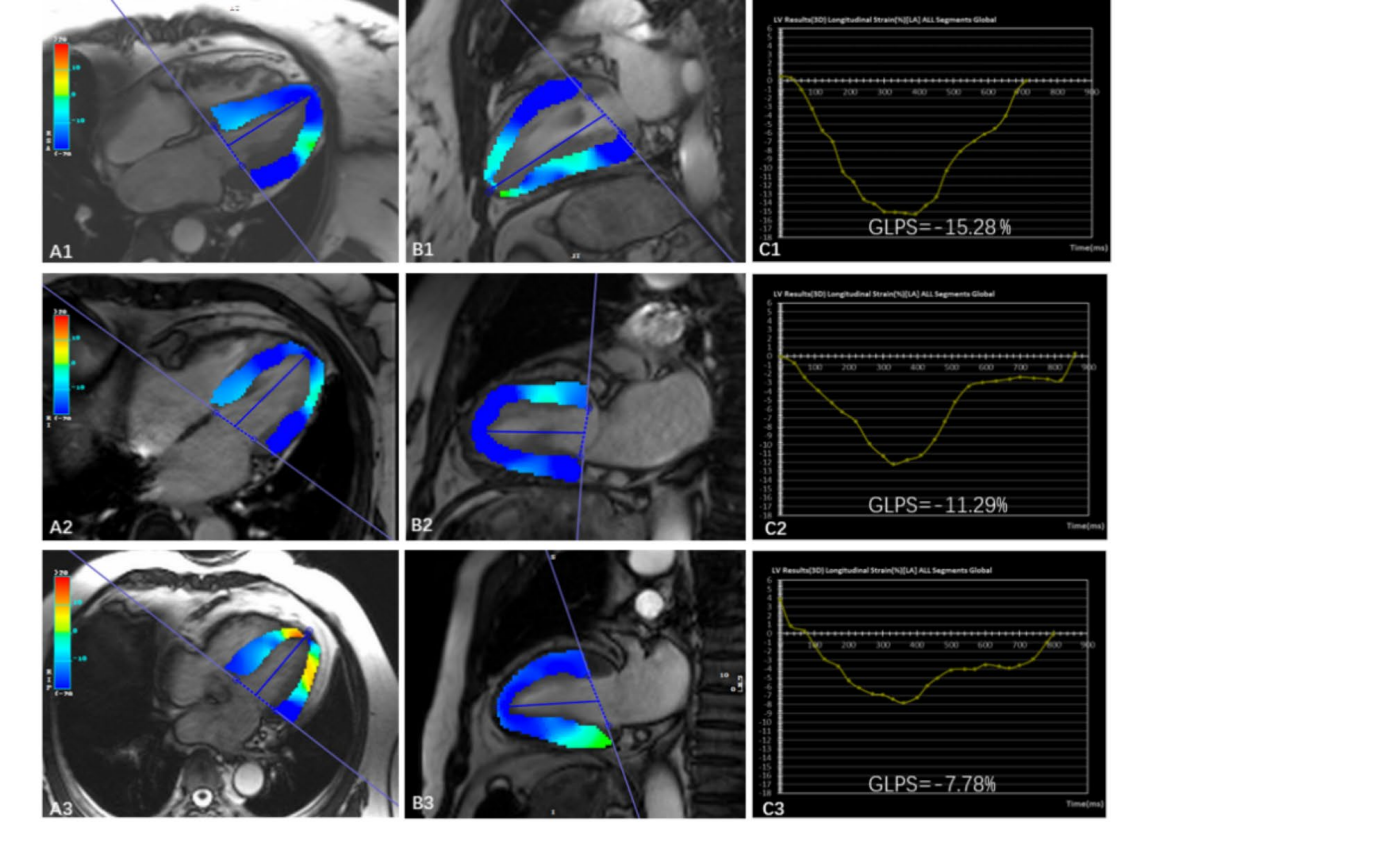
LV pseudo-color images of long-axis four- and two-chambers CMR cine images at the end-systole (**A**,**B**) and corresponding GLPS curves(**C**) in three groups of MetS. **A1-C1.** 45-year-old female MetS patient without CAS. **A2-C2.** 73-year-old female MetS patient with NOCAS. **A3-C3.** 57-year-old male MetS patient with OCAS

The MetS patients were further stratified based on the degree of CAS, comprising MetS(CAS−), MetS(NOCAS+) (n = 30, mean age 58 ± 8 years, 23 males), and MetS(OCAS+) (n = 54, mean age 61 ± 9 years, 47 males). Compared with the MetS(CAS−) group, the MetS(NOCAS+) and MetS(OCAS+) groups showed higher rates of hypertension(63.83% vs. 100.00% and 63.83% vs. 88.89%, respectively, p < 0.05) (Table S1 in the Supplementary material). Compared to the MetS(CAS−) and MetS(NOCAS+) groups, the MetS(OCAS+) group showed significantly increased LV-EDVi, LV-ESVi, and decreased LVEF, LVGFI (all p < 0.05), Table 3. Compared to MetS(CAS−), MetS(NOCAS+) group showed increased LVMi (61.94 ± 18.93 g/m^2^ vs. 48.13 ± 16.03 g/m^2^, p < 0.05), MetS(OCAS+) group showed increased LVMi (64.69 ± 22.70 g/m^2^ vs. 48.13 ± 16.03 g/m^2^, p < 0.05) and decreased LV-SVi (38.86 ± 11.64 mL/m^2^ vs. 46.02 ± 14.80 mL/m^2^, p < 0.05), Fig. 1. In terms of LV deformation parameters, GLPS were decreased gradually from MetS(CAS−) through MetS(NOCAS+) to MetS(OCAS+) (− 13.02 ± 2.32% vs. − 10.34 ± 4.05% vs. − 7.55 ± 4.48%, all p < 0.05), Fig. 2. LV GRPS, GCPS, PSSR and PDSR in radial and circumferential directions were decreased in MetS(OCAS+) compared to both MetS(NOCAS+) and MetS(CAS−)groups (all p < 0.05). PSSR and PDSR in the longitudinal direction showed a decrease in the MetS(OCAS+) compared to the MetS(CAS−) (all p < 0.05). There was a significantly higher proportion of subendocardial LGE in the MetS(OCAS+) than MetS(NOCAS+) group (31.48% vs. 3.33%; p < 0.05).

**Table 3:** Comparison of LV function and strain parameters among MetS (CAS−), MetS (NOCAS+) and MetS (OCAS+)

	MetS		
	CAS− (n = 47)	NOCAS+ (n = 30)	OCAS+ (n = 54)
LV-EDVi, mL/m^2^	72.49 ± 22.21	74.87 ± 21.41	98.08 ± 43.06^§#^
LV-ESVi, mL/m^2^	26.46 ± 11.86	30.56 ± 21.52	59.22 ± 44.24^§#^
LV-SVi, mL/m^2^	46.02 ± 14.80	44.31 ± 9.33	38.86 ± 11.64^§^
LVMi, g/m^2^	48.13 ± 16.03	61.94 ± 18.93^§^	64.69 ± 22.70^§^
LVEF, %	63.85 ± 11.72	62.59 ± 15.48	46.51 ± 19.42^§#^
LVGFI	49.02 ± 11.10	42.40 ± 12.20	32.76 ± 14.86^§#^
LV GPS, %			
GRPS, %	32.03 ± 9.61	27.72 ± 11.31	18.39 ± 11.59^§#^
GCPS, %	− 18.99 ± 5.41	− 17.76 ± 4.49	− 13.65 ± 6.42^§#^
GLPS, %	− 13.02 ± 2.32	− 10.34 ± 4.05^§^	− 7.55 ± 4.48^§#^
PSSR(1/s)			
PSSR-R	2.15 ± 0.94	1.84 ± 0.94	1.10 ± 0.94^§#^
PSSR-C	− 1.09 ± 0.39	− 1.02 ± 0.23	− 0.71 ± 0.48^§#^
PSSR-L	− 0.80 ± 0.44	− 0.62 ± 0.44	− 0.43 ± 0.81^§^
PDSR(1/s)			
PDSR-R	− 2.09 ± 1.01	− 1.86 ± 0.98	− 1.17 ± 1.46^§#^
PDSR-C	1.16 ± 0.41	0.98 ± 0.19	0.77 ± 0.33^§#^
PDSR-L	0.69 ± 0.54	0.63 ± 0.37	0.50 ± 0.32^§^
LGE pattern, n (%)			
None	–	18 (60.00%)	21 (38.89%)
LGE+	–	12 (40.00%)	33 (61.11%)
Subendocardial	–	1 (3.33%)	17 (31.48%)^#^
Midmyocardium	–	3 (10.00%)	9 (16.67%)
Subepicardial	–	2 (6.67%)	3 (5.56%)
Transmural	–	6 (20.00%)	7 (12.96%)

All values are presented as n (%), mean ± standard deviation

Abbreviations as listed in Table 2. LGE, late gadolinium enhancement

^§^: p < 0.05 vs. MetS (CAS−) group

^#^: p < 0.05 vs. MetS (NOCAS+) group

### Determinants of impaired LV function and deformation in MetS patients

Univariable and multivariable linear regression analyses were performed for LV GRPS, GCPS, and GLPS (Table 4). The results of multivariable linear regression analysis showed that the degree of CAS was independently associated with impaired LV GRPS (β =  − 0.289, p < 0.001) and GLPS (β = 0.254, p = 0.004). Furthermore, Male and NT‑proBNP were independently associated with GPS in all three directions (all p < 0.05). LGE+ was independently associated with GCPS (β = 0.212, p = 0.014) and GLPS (β = 0.185, p = 0.037). Determinants of impaired LV function and LV PSSR and PDSR in MetS patients are presented in Tables S2-5 of Supplementary material. The degree of CAS was an independent determinant of PSSR-S (β =  − 0.584, p < 0.05), PSSR-C (β = 0.304, p < 0.05), and PDSR-C (β =  − 0.223, p < 0.05).

**Table 4:** Determinants of impaired LV deformation in MetS patients

	GRPS, %				GCPS, %				GLPS, %			
	Univariable		Multivariable		Univariable		Multivariable		Univariable		Multivariable	
	γ	p	β	p	γ	p	β	p	γ	p	β	p
degree of CAS	− 0.483	< 0.001	− 0.289	< 0.001	0.383	< 0.001			0.540	< 0.001	0.254	0.004
Gensini score	− 0.354	< 0.001			0.371	< 0.001			0.455	< 0.001		
Age	− 0.072	0.417			0.127	0.147			0.114	0.194		
Male	− 0.245	0.005	− 0.159	0.033	0.215	0.014	0.178	0.024	0.252	0.004	0.171	0.019
BMI	0.402	< 0.001			− 0.310	< 0.001			− 0.482	< 0.001		
Hypertension	− 0.114	0.194			0.069	0.433			0.143	0.103		
T2DM	− 0.066	0.455			0.062	0.485			0.053	0.544		
HbA1c	0.079	0.443			− 0.065	0.529			− 0.154	0.134		
TG	0.265	0.003			− 0.266	0.002			− 0.277	0.002		
HDL	− 0.260	0.003			0.177	0.048			0.334	< 0.001		
NT-proBNP	− 0.518	< 0.001	− 0.409	< 0.001	0.527	< 0.001	0.444	< 0.001	0.532	< 0.001	0.368	< 0.001
LGE+	0.419	< 0.001			− 0.403	< 0.001	0.212	0.014	0.488	< 0.001	0.185	0.037

Abbreviations as listed in Tables 1, 2

NT-proBNP was log-transformed before being included in the regression analysis

**Fig. 3:**
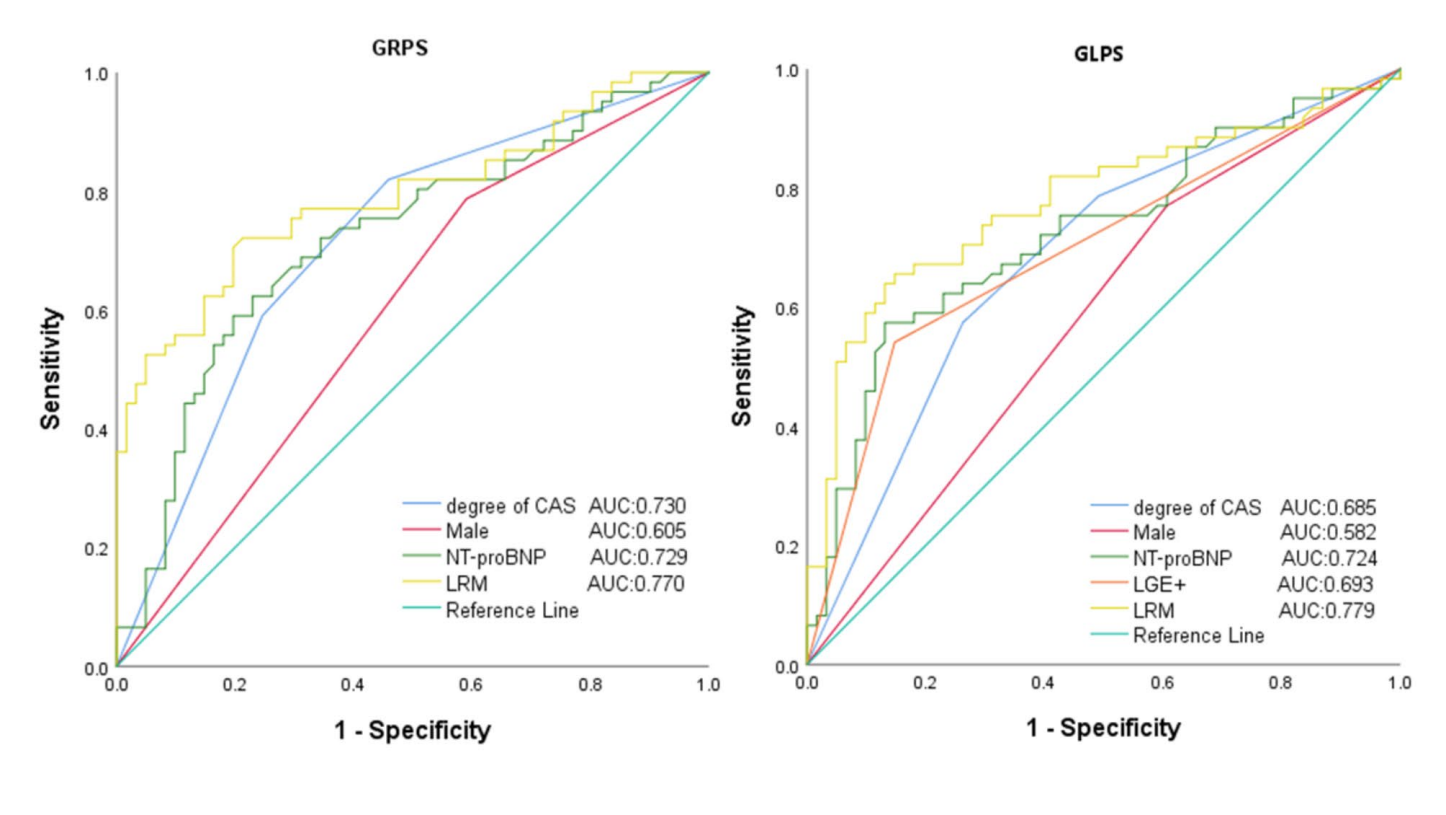
Receiver operating characteristic curve (ROC) for predicting the impaired left ventricle global radial peak strain (LV GRPS) and impaired left ventricle global longitudinal peak strain (GLPS) of the damaged. AUC, Area under the curve

The results of ROC analysis for the prediction of impaired GRPS showed that the AUC of the degree of CAS was 0.730 (sensitivity, 0.831; specificity, 0.545). The AUC of LRM constructed by the combining degree of CAS was 0.770(sensitivity, 0.705; specificity, 0.770). The prediction of impaired GLPS showed that the AUC of degree of CAS was 0.685 (sensitivity, 0.574; specificity, 0.738). The AUC of LRM constructed by combining the degree of CAS was 0.779 (sensitivity, 0.639; specificity, 0.869), Fig. 3.

### Reproducibility of LV function and strain parameters

Intra-observer correlation coefficients for LV function and strain parameters ranged from 0.829 to 0.989. Inter-observer correlation coefficients for these parameters ranged from 0.856 to 0.985, demonstrating excellent reproducibility. Detailed results are shown in Table S6 of the supplementary material.

## Discussion

This study investigated differences in LV function and deformation between MetS patients with and without CAS, explored the LV dysfunction, deformation, and LGE patterns of MetS patients with varying degrees of CAS, and identified determinants. The main findings are as follows: (1) the presence of CAS further deteriorated LV function, LV GPS, LV PSSR, and PDSR in MetS patients; (2) impairment of LV GLPS and increased LVMi were shown in MetS(NOCAS+) patients, impaired LV function, LV GPS in all three directions, LV PSSR and PDSR in the radial and circumferential directions were shown in MetS(OCAS+) patients; (3) the degree of CAS was an independent factor for both radial and longitudinal reduction of GPS in all MetS patients. Increased CAS severity worsens LV function and strain in MetS patients, with a degree of CAS being an independent determinant of impaired LV strain. Early detection and management of MetS patients with varying degrees of CAS are crucial to prevent further cardiac deterioration.

### MetS impact on LV function and deformation

Our CMR study showed that LV GRPS and GLPS were reduced, while GCPS was preserved, and PDSR was reduced in all three directions in MetS(CAS−) compared to controls. Previous echocardiography studies [13, 25] have shown that MetS is independently associated with LV diastolic dysfunction and impaired GLPS. Reductions in GRPS and GLPS have also been demonstrated [26]. The potential mechanism may be that each component of MetS independently influences cardiac structure and function, and their combination carries an additional compounded risk [27]. Specifically, adipose tissue deposition increases free fatty acids, angiotensinogen, resistin, and inflammatory cytokines, damaging endothelial function and leading to coronary microvascular dysfunction. This dysfunction raises a vicious cycle with increased resistance and blood pressure in peripheral blood vessels and exacerbates myocardial dysfunction by reducing insulin-mediated glucose uptake [28, 29]. Moreover, early ischemic myocardial dysfunction linked to coronary microvascular dysfunction is evidenced by an impairment in LV diastolic function initially, which is a hallmark of myocardial dysfunction in patients with MetS [30]. Therefore, even in the absence of definitive cardiovascular disease, MetS individuals often show adverse cardiac remodeling and myocardial dysfunction.

### Aggravated effect of varying degrees of CAS on LV remodeling in MetS patients

Coronary atherosclerosis in the MetS population deserves attention not only because of its high prevalence rate [31]. Chronically exposed to hyperglycemia and associated atherosclerotic abnormalities, such as hyperlipidemia and hypertension, led to subjects with MetS not only having more extensive CAS but also showing faster CAD progression than subjects without MetS [32, 33]. Elevated blood pressure is one of the main components of MetS. Pressure overload of the LV results in a compensatory increment in LVM, initially causing increased wall thickness. Moreover, CAS causes insufficient myocardial blood flow, especially under pressure. These hypertrophy and remodeling provide an adaptive mechanism for maintaning normal LV systolic wall stress under increased blood pressure and myocardial ischemia [34, 35]. Hypertrophy is a hallmark of MetS-related cardiac remodeling, which has been confirmed in previous studies [36]. Our study found that MetS(CAS+) had significantly higher LVM than MetS(CAS−), and even in MetS(NOCAS+), LVM was significantly increased. In addition to considering the higher proportion of hypertension in MetS(CAS+), it is also necessary to account for the exacerbating effect of NOCAS on myocardial hypertrophy in the complex pathological conditions of MetS. Thus, even NOCAS has an aggravating effect on LV remodeling in MetS patients.

### OCAS aggravates LV function in MetS patients

Our findings showed that there was no difference in LV-SVi between the MetS (CAS−) groups and MetS(NOCAS+) group, but in the MetS(OCAS+) group, LV-SVi significantly decreased compared with MetS(CAS−). The combination of OCAS exacerbates the increase of LV-EDVi, LV-ESVi, and the decrease of LVEF and LVGFI in MetS patients. As with previous studies, LV-SV does not significantly decrease in MetS patients, while OCAS aggravated LV dysfunction and remodeling in MetS patients [13, 25, 37]. This is probably because pathological co-development exists between structural remodeling and LV-SV. CAS-induced remodeling affects the passive-elastic properties and LVM, and pathologically mediates LV-SV in the mid and long-term after remodeling [34]. When oxygen supply–demand in the LV myocardial area supplied by the stenotic vessels is imbalanced, regional dysfunction is caused, resulting in the decrease of LV-SV [38]. Additionally, OCAS decreases coronary artery flow, further exacerbating myocardial dysfunction through ischemia-related dysfunction, ventricular remodeling, excessive neurohumoral stimulation, and abnormal calcium cycling in myocardial cells [39]. Thus, LV-SV was significantly impaired in MetS patients with OCAS, and as the degree of CAS increases, the LV function impairment in MetS patients and the degree of reconstruction becomes progressively more severe.

### Exacerbated impact on LV deformation in MetS patients with varying degrees of CAS

Our study showed that MetS(NOCAS+) showed a significant decrease in GLPS compared to MetS(CAS−). Compared to MetS(CAS−) and MetS(NOCAS+), MetS(OCAS+) showed significantly decreased GPS in three directions. Previous echocardiography study have indicated that the LV myocardium strains decrease with increasing CAS severity, with NOCAS patients showing significantly lower global longitudinal peak strain (GLPS) in mild stenosis (25% < stenosis ≤ 50%) compared to those with slight stenosis (stenosis ≤ 25%) [40]. GLPS generally reflects the contraction of endocardial and epicardial fibers, GRPS reflects the endocardial fibers, and GCPS reflects the mid-myocardium fibers. GLPS, among them, is aligned with endocardial fibers where ischemia first develops and is theoretically the most sensitive parameter for detecting myocardial abnormalities because of myocardial ischemia [41]. Previous study point out that while coronary microvascular dysfunction might not significantly reduce GLPS in NOCAS patients, the aggravation of this dysfunction is an essential reason for the sudden increase of ischemia and the resulting reduction of GLPS [42]. Moreover, NOCAS patients are in a complex pathological context where MetS contributes to the development of CAD, exacerbating endocardial ischemia and further damaging LV deformation. Therefore, deformations of LV myocardium in MetS decreased on different levels along with the aggravation of CAS.

### Clinical parameters associated with decreased LV deformation in MetS patients

A higher prevalence of male patients in MetS(CAS+) was shown in our study, with an independent association between male sex and impaired LV deformation. Similar findings in the Framingham Heart Study indicated that males with MetS were more susceptible to developing CAD [43]. NT‑proBNP increased significantly with the severity of CAS in MetS patients and demonstrated excellent performance in predicting impaired GRPS and GLPS. NT‑proBNP is a neurohormone released by cardiomyocytes in response to increased ventricular wall stress, hypertrophy, and volume overload [44]. Moreover, the degree of CAS was independently correlated with impaired GRPS and GLPS after univariable and multivariable analysis. As an independent determinant, the degree of CAS performs well in predicting impaired GRPS and GLPS, when combined with other influencing factors to construct LRM, further improves diagnostic efficacy. Beyond traditional biochemical indicators, the degree of CAS, as a cardiovascular risk factor with rising incidence, warrants greater attention in the clinical management of cardiac function impairment in MetS patients. Early intervention and control of the progression of CAS in MetS patients will help reduce the occurrence of adverse cardiovascular and cerebrovascular events.

### Limitations

Our study also has several limitations. Firstly, as a retrospective single-center study, it is subject to patient selection bias. Secondly, BMI was used as a substitute for clinical waist circumference data to reflect central obesity, and the practicability has been confirmed [17]. Thirdly, the combination and number of cardiovascular risk factors contributing to MetS need further exploration in future studies to fully understand the additive effect of CAS on myocardial damage in MetS patients. Finally, we did not evaluate the microvascular dysfunction and fractional flow reserve in the study. These limitations underscore the need to carefully interpret our results and suggest that further prospective, multi-center studies are necessary to confirm and expand upon our findings.

## Conclusion

This study demonstrated that NOCAS exacerbates GLPS reduction and LVMi increase in MetS patients. As CAS severity increases, LV function and deformation worsen, and the degree of CAS independently affects impaired GRPS and GLPS in MetS patients. Besides traditional biochemical indicators, incorporating CAS assessment and CMR assessment of the LV into routine evaluations ensures a more holistic approach to managing MetS patients. Timely intervention of CAS is crucial for improving cardiovascular outcomes in this high-risk population.

## Electronic supplementary material

Below is the link to the electronic supplementary material.

Supplementary Material

## Data Availability

The datasets used and analyzed during the current study are available from the corresponding author on reasonable request.

## Abbreviations

MetS: Metabolic syndrome
CAD: Coronary artery disease
CAS: Coronary artery stenosis
LV: Left ventricular
MACE: Major adverse cardiovascular events
NOCAS: Non-obstructive coronary artery stenosis
CMR: Cardiac magnetic resonance
GPS: Global peak strain
WC: Waist circumference
T2DM: Type 2 diabetes mellitus
OCAS: Obstructive coronary artery stenosis
LM: Left main
LAD: Left anterior descending
LCX: Left circumflex
RCA: Right coronary artery
LGE: Late gadolinium enhancement
EDV: End-diastolic volume
ESV: End-systolic volume
EF: Ejection fraction
SV: Stroke-volume
M: Mass
i: Index
LVGFI: Left ventricular global function index
GRPS: Global radial peak strain
GCPS: Global circumferential peak strain
GLPS: Global longitudinal peak strain
PSSR: Peak systolic strain rate
PDSR: Peak diastolic strain rate
