# Supplementary material for: Assessing coronary artery stenosis exacerbated impact on left ventricular function and deformation in metabolic syndrome patients by 3.0 T cardiac magnetic resonance imaging
Source: Cardiovasc Diabetol. 2024 Nov 18;23:414. doi: 10.1186/s12933-024-02492-9 (PMC11575079; doi:10.1186/s12933-024-02492-9)
Supplement: Supplementary file 1 — Supplementary Material [file 12933_2024_2492_MOESM1_ESM.docx]

**Table S1 Demographic and clinical characteristics among the MetS(CAS−), MetS(NOCAD+) and MetS(OCAD+) groups**

|  | | MetS | | |
| --- | --- | --- | --- | --- |
|  |  | CAS− (n= 47) | NOCAS+ (n=30) | OCAS+ (n=54) |
| Baseline characteristics | |  |  |  |
|  | Age (years) | 52 ± 14 | 58 ± 8 | 61 ± 9^§^ |
|  | Male, n (%) | 26(55.32%) | 23(76.67%) | 42(77.78%) |
|  | BMI (kg/m^2^) | 26.26 ± 2.93 | 27.01 ± 3.47^§^ | 26.14 ± 2.87^§^ |
|  | Smoking, n (%) | 14(29.79%) | 9(30.00%) | 18(33.33%) |
|  | SBP (mmHg) | 133 ± 23 | 138 ± 26 | 137 ± 18 |
|  | DBP (mmHg) | 84 ± 18 | 84 ± 16 | 84 ± 13 |
|  | HR, min^-1^ | 75 ± 16 | 77 ± 12 | 77 ± 16 |
| Cardiovascular risk (n, %) | |  |  |  |
|  | Hypertension, n (%) | 30(63.83%) | 30(100.00%) ^§^ | 48(88.89%) ^§^ |
|  | T2DM, n (%) | 16(34.04%) | 14(46.67%) | 33(61.11%) |
|  | Dyslipidemia | 44(93.62%) | 27(90.00%) | 46(85.19%) |
|  | Obesity, n (%) | 32(68.09%) | 20(66.67%) | 36(66.67%) |
| Laboratory parameters | |  |  |  |
|  | HbA1c (%) | 6.40 ± 0.87 | 6.70 ± 1.18^§^ | 7.72 ± 1.35^§^ |
|  | TG, mmol/L | 2.74 ± 1.76 | 2.47 ± 1.54^§^ | 2.67 ± 2.26^§^ |
|  | TC, mmol/L | 4.26 ± 1.19 | 4.28 ± 1.16 | 3.79 ± 1.07 |
|  | HDL, mmol/L | 0.84 ± 0.27 | 0.91 ± 0.19^§^ | 0.93 ± 0.28^§^ |
|  | LDL, mmol/L | 2.28 ± 0.88 | 2.53 ± 0.85 | 2.07 ± 0.86 |
|  | Troponin,ng/L | 70.30 ± 166.08 | 218.66 ± 478.78 | 114.31 ± 346.46 |
|  | NT‑proBNP | 444.27 ± 843.91 | 712.26 ± 1122.79^§^ | 1580.51 ± 2285.79^§^ |
|  | eGFR (ml/min/1.73m^2^) | 77.20 ± 28.66 | 78.29 ± 23.07 | 70.41 ± 21.82 |
| Concomitant medication, n (%) | |  |  |  |
|  | Insulin | 5(10.64%) | 1(3.33%) | 12(22.22%)^#^ |
|  | Biguanides | 2(4.26%) | 6(20.00%) | 13(24.07%)^§^ |
|  | ACEI/ARB | 12(25.53%) | 11(36.67%) | 24(44.44%) |
|  | Satins | 4(8.51%) | 7(23.33%) | 15(27.78%)^§^ |
|  | Aspirin | 3(6.38%) | 8(26.67%)^§^ | 16(29.63%)^§^ |
| Coronary artery related parameters | |  |  |  |
|  | Number of coronary arteries affected  One/Two/Three-vessel | − | 7(23.33%)/17(56.67%)/6(20.00%) | 8(14.81%)/4(7.40%)/42(84.00%) |
|  | Location of coronary artery occlusion  (RCA /LMCA/LAD/LCX) | − | − | 31(62.00%)/6(12.00%)/44(88.00%)/31(62.00%) |
|  | Most severe stenosis (%) | 0.00±0.00 | 29.69±15.51^§^ | 88.98±13.55^§#^ |
|  | Gensini score | 0.00±0.00 | 9.00±6.98^§^ | 67.21±51.69^§#^ |

All values are presented as n (%), mean ± standard deviation. BMI, body mass index; SBP, systolic blood pressure; DBP, diastolic blood pressure; HR, heart rate; T2DM, type 2 diabetes mellitus; HbA_1_c, glycated haemoglobin; TG, triglyceride; TC, total cholesterol; HDL-C, High density lipoprotein cholesterol; LDL-C, Low density lipoprotein cholesterol; NT‑proBNP, amino-terminal pro-B-type natriuretic peptide; eGFR, estimated glomerular filtration rate; ACEI, angiotensin converting enzyme inhibitor; ARB, angiotensin receptor blocker.

^§^: p < 0.05 vs. MetS (CAS−) group.

^#^: p < 0.05 vs. MetS (NOCAS+) group.

**Table S2 Determinants of impaired LV PSSR in MetS patients**

|  | PSSR-S(1/s) | |  | PSSR-C(1/s) | |  | PSSR-L(1/s) | |
| --- | --- | --- | --- | --- | --- | --- | --- | --- |
|  | Univariable | Multivariable |  | Univariable | Multivariable |  | Univariable | Multivariable |
|  | γ | β |  | γ | β |  | γ | β |
| degree of CAS | −0.439^※^ | −0.584^※^ |  | 0.382^※^ | 0.304^※^ |  | 0.254^※^ |  |
| Gensini score | −0.339^※^ |  |  | 0.315^※^ |  |  | 0.162 |  |
| Age | −0.132 |  |  | 0.197^※^ |  |  | 0.081 |  |
| Male | −0.103 |  |  | 0.104 |  |  | 0.119 |  |
| BMI | 0.189^※^ |  |  | −0.270 |  |  | −0.223^※^ |  |
| Hypertension | −0.089 |  |  | 0.081 |  |  | 0.091 |  |
| T2DM | −0.114 |  |  | 0.098 |  |  | 0.011 |  |
| HbA1c | −0.062 |  |  | −0.065 |  |  | −0.241^※^ |  |
| TG | 0.212^※^ |  |  | −0.271^※^ |  |  | −0.193^※^ |  |
| HDL | −0.193^※^ | 0.274^※^ |  | 0.141 |  |  | 0.185^※^ |  |
| NT‑proBNP | −0.401^※^ | −0.213^※^ |  | 0.341^※^ | 0.240※ |  | 0.224^※^ |  |
| LGE+ | −0.306^※^ |  |  | 0.296^※^ |  |  | 0.346^※^ | 0.353^※^ |

Abbreviations as listed in Table S1.

^※^: p < 0.05

**Table S3 Determinants of impaired LV PDSR in MetS patients**

|  | PDSR-S(1/s) | |  | PDSR-C(1/s) | |  | PDSR-L(1/s) | |
| --- | --- | --- | --- | --- | --- | --- | --- | --- |
|  | Univariable | Multivariable |  | Univariable | Multivariable |  | Univariable | Multivariable |
|  | γ | β |  | γ | β |  | γ | β |
| degree of CAS | 0.317^※^ |  |  | −0.450^※^ | −0.223^※^ |  | −0.196 |  |
| Gensini score | 0.220^※^ |  |  | −0.365^※^ |  |  | −0.171 |  |
| Age | 0.116 |  |  | −0.333^※^ |  |  | −0.052 |  |
| Male | 0.237^※^ | 0.228^※^ |  | −0.122 |  |  | −0.050 |  |
| BMI | 0.245^※^ |  |  | 0.371^※^ |  |  | 0.191^※^ | 0.191^※^ |
| Hypertension | 0.078 |  |  | −0.203^※^ |  |  | −0.096 |  |
| T2DM | −0.005 |  |  | −0.169 |  |  | 0.007 |  |
| HbA1c | −0.003 |  |  | 0.072 |  |  | −0.016 |  |
| TG | −0.203^※^ |  |  | 0.403^※^ | 0.205^※^ |  | 0.110 |  |
| HDL | 0.121 |  |  | −0.251^※^ |  |  | −0.095 |  |
| NT‑proBNP | 0.302^※^ | 0.293^※^ |  | −0.455^※^ | -0.356^※^ |  | −0.130 |  |
| LGE+ | 0.225^※^ |  |  | −0.368^※^ |  |  | −0.186^※^ |  |

Abbreviations as listed in Table S1.

^※^: p < 0.05

**Table S4 Determinants of impaired LV function in MetS patients**

|  | LV-EDVi, mL/m^2^ | |  | LV-ESVi, mL/m^2^ | |  | LV-SVi, mL/m^2^ | |
| --- | --- | --- | --- | --- | --- | --- | --- | --- |
|  | Univariable | Multivariable |  | Univariable | Multivariable |  | Univariable | Multivariable |
|  | γ | β |  | γ | β |  | γ | β |
| degree of CAS | 0.329^※^ |  |  | 0.419^※^ | 0.245^※^ |  | −0.246^※^ |  |
| Gensini score | 0.297^※^ |  |  | 0.351^※^ |  |  | −0.149 |  |
| Age | −0.002 |  |  | 0.092 |  |  | −0.253^※^ | -0.273^※^ |
| Male | 0.180^※^ | 0.206^※^ |  | 0.179^※^ |  |  | 0.002 |  |
| BMI | −0.246^※^ |  |  | −0.312^※^ |  |  | 0.181^※^ |  |
| Hypertension | 0.050 |  |  | 0.079 |  |  | −0.080 |  |
| T2DM | 0.060 |  |  | 0.079 |  |  | −0.052 |  |
| HbA1c | −0.150 |  |  | −0.168 |  |  | 0.079 |  |
| TG | −0.215^※^ |  |  | 0.023 |  |  | 0.114 |  |
| HDL | 0.101 |  |  | 0.156 |  |  | −0.153 |  |
| NT‑proBNP | 0.553^※^ | 0.563^※^ |  | 0.577^※^ | 0.489^※^ |  | −0.088 |  |
| LGE+ | 0.306^※^ |  |  | 0.354^※^ |  |  | -0.133 |  |

Abbreviations as listed in Table S1.

^※^: p < 0.05

**Table S5 Determinants of impaired LV function in MetS patients**

|  | LVMi, g/m^2^ | |  | LVEF, % | |  | LVGFI | |
| --- | --- | --- | --- | --- | --- | --- | --- | --- |
|  | Univariable | Multivariable |  | Univariable | Multivariable |  | Univariable | Multivariable |
|  | γ | β |  | γ | β |  | γ | β |
| degree of CAS | 0.341^※^ |  |  | −0.425^※^ | −0.257^※^ |  | −0.481^※^ |  |
| Gensini score | 0.246^※^ |  |  | −0.367^※^ |  |  | −0.368^※^ |  |
| Age | −0.004 |  |  | −0.163 |  |  | −0.165 |  |
| Male | 0.355^※^ | 0.474^※^ |  | −0.133 |  |  | −0.256^※^ |  |
| BMI | −0.360^※^ |  |  | 0.309^※^ |  |  | 0.411^※^ |  |
| Hypertension | 0.288^※^ | 0.171^※^ |  | −0.095 |  |  | −0.228^※^ |  |
| T2DM | −0.005 |  |  | −0.066 |  |  | −0.051 |  |
| HbA1c | −0.209^※^ |  |  | 0.128 |  |  | 0.214^※^ |  |
| TG | −0.307 |  |  | 0.262^※^ |  |  | 0.337^※^ |  |
| HDL | 0.210^※^ |  |  | −0.154 |  |  | −0.251^※^ |  |
| NT‑proBNP | 0.510^※^ | 0.533^※^ |  | −0.538^※^ |  |  | −0.529^※^ |  |
| LGE+ | 0.0380^※^ |  |  | −0.339^※^ | −0.257^※^ |  | −0.404^※^ |  |

Abbreviations as listed in Table S1.

^※^: p < 0.05

**Table S6 Intra- and inter-observer reproducibility for left ventricular function and strain parameters**

|  | | Intra-observer correlation coefficients (95% CI) | Inter-observer correlation coefficients (95% CI) |
| --- | --- | --- | --- |
| Function parameters | |  |  |
| LV-EDVi | | 0.989 (0.982,0.994) | 0.985 (0.973,0.991) |
| LV-ESVi | | 0.987 (0.977,0.992) | 0.977 (0.959,0.987) |
| LV-SVi | | 0.921 (0.865,0.954) | 0.915 (0.855,0.951) |
| LVMi | | 0.969 (0.946,0.983) | 0.955 (0.921,0.975) |
| LVEF | | 0.946 (0.907,0.969) | 0.942 (0.900,0.967) |
| LVGFI | | 0.935 (0.885,0.963) | 0.917 (0.857,0.953) |
| LV GPS | |  |  |
|  | GRPS | 0.923 (0.868,0.956) | 0.880 (0.798,0.930) |
|  | GCPS | 0.947 (0.909,0.970) | 0.954 (0.921,0.974) |
|  | GLPS | 0.880 (0.786,0.932) | 0.890 (0.897,0.967) |
| PSSR | |  |  |
|  | PSSR-S | 0.944 (0.904,0.968) | 0.924 (0.870,0.945) |
|  | PSSR-C | 0.837 (0.729,0.904) | 0.856 (0.760,0.916) |
|  | PSSR-L | 0.927 (0.876,0.958) | 0.931 (0.882,0.960) |
| PSDR | |  |  |
|  | PSDR-S | 0.948 (0.910,0.970) | 0.934 (0.886,0.962) |
|  | PSDR-C | 0.838 (0.733,0.905) | 0.876 (0.779,0.930) |
|  | PSDR-L | 0.829 (0.718,0.899) | 0.864 (0.773,0.920) |

All values are presented as median (interquartile range). The “−” indicates the direction of strains. LV, left ventricular; EDV, end-diastolic volume; ESV, end-systolic volume; EF, ejection fraction; SV, stroke-volume; M, mass; i: index; LVGFI, left ventricular global function index; GRPS, global radial peak strain; GCPS, global circumferential peak strain; GLPS, global longitudinal peak strain; PSSR, peak systolic strain rate; PDSR, peak diastolic strain rate.
